# Glycemic control in children and adolescents with type 1 diabetes: association with parental diabetes distress and anxiety

**DOI:** 10.3389/fendo.2026.1853376

**Published:** 2026-07-29

**Authors:** Laila AlBishi

**Affiliations:** Department of Pediatrics, Faculty of Medicine,Diabetes and Chronic Diseases Unit, University of Tabuk, Tabuk, Saudi Arabia

**Keywords:** anxiety, glycated hemoglobin, glycemic control, parental distress, Saudi Arabia, type 1 diabetes

## Abstract

**Background:**

Managing type 1 diabetes (T1D) in children is an overwhelming challenge for both the family and parents, whose emotional health can affect the child’s glycemic levels. Distress/Anxiety that parents experience related to their child's T1D has been identified as an increasing factor that affects the child's ability to manage their T1D and clinical outcomes. The purpose of this study was to assess the relationship between Glycemic Control and Parental Diabetes Related Stress/Anxiety in a Saudi Arabian Cohort.

**Methods:**

A cross-sectional study was conducted with 87 parents of children and adolescents with T1D who visited the Pediatric Diabetes Specialty Clinic. Parental diabetes-related emotional experiences and anxiety levels were measured using valid instruments; specifically, the Parent Diabetes Distress Scale and the State-Trait Anxiety Inventory. The association between variables was determined through correlational analysis and by comparing across levels of glycemic control using HbA1c values. Additionally, demographic/clinical characteristics related to glycemic control were examined in relation to other characteristics.

**Results:**

The average HbA1c of participants indicated suboptimal glycemic control (mean = 8.79%). There were no differences in overall diabetes-related distress across the different glycemic ranges. The subscale of parental distress related to teen management and the relationship between the parent/child were both positively correlated to an individual's glycemic control. Older age and longer duration of the disease also correlated with lower levels of glycemic control.

**Conclusion:**

Psychosocial evaluation and family-focused interventions may be useful as standard diabetes management practices that lead to better emotional and metabolic health outcomes. Parental distress, parental anxiety, and suboptimal glycemic control are closely related. The findings support the need to assess the parents’ mental/psychological state as part of an overall management strategy for pediatric diabetes, especially with limited regional evidence.

## Introduction

Type 1 diabetes (T1D) is a major autoimmune endocrine disorder that predominantly affects pediatric populations. It is estimated that more than 1.2 million children and adolescents (aged ≤20 years) worldwide are affected by T1D. Its presentation varies across races, countries, and regions (1). Management of T1D involves insulin therapy, dietary management, physical activity, and psychosocial support (2).

Glycated hemoglobin (HbA1c) is traditionally considered the gold standard for monitoring long-term glycemic control in diabetes (3). HbA1c provides an objective measure of average blood glucose levels over the preceding 2–3 months (4). It is also used to assess long-term glycemic variability (5), which has been associated with both macrovascular and microvascular complications, including nephropathy, neuropathy, and retinopathy (6). Achieving optimal HbA1c levels is essential to reduce the risk of acute complications such as severe hypoglycemia and diabetic ketoacidosis, particularly in children and adolescents. Advances in diabetes care, including continuous glucose monitoring (CGM), connected insulin pens, and patient education, have been shown to improve glycemic control (7). While the traditional metric for assessing long-term control is glycated hemoglobin (HbA1c), growing evidence supports the use of Time in Range (TIR) as an additional assessment tool, which expresses the proportion of time that measurements are within a given target range and thus gives an impression of glycemic variability in a more detailed manner than the HbA1c value alone (8). Further, TIR may be more closely associated with patient and caregiver experiences, including treatment burden and psychological stress, as it captures real-time glycemic excursions that often require immediate attention (9). Consequently, greater glycemic variability and reduced TIR may contribute to heightened parental anxiety due to the need for constant monitoring and intervention (10).

Despite these advances, achieving recommended glycemic targets in children and adolescents remains challenging. International guidelines, including those of the American Diabetes Association and the International Society for Pediatric and Adolescent Diabetes (ISPAD), indicate that optimal glycemic control is often not achieved in this population (11). This suboptimal control is attributed to a combination of physiological, psychological, and social factors that collectively influence disease management.

During adolescence, rapid endocrine changes can induce insulin resistance and increase glycemic variability, necessitating frequent adjustments in insulin therapy (12). In younger children, dependence on caregivers for disease management adds further complexity. Parents play a central role in managing their child’s condition from diagnosis through ongoing treatment adherence, and this responsibility is frequently associated with diabetes-related distress (13).

Parental responsibility for diabetes management may lead to persistent stress and worry, resulting in exhaustion, sleep disturbances, and impaired daily functioning (14). Parental diabetes-related distress (PDD) is defined as the negative emotional burden experienced by parents of children with T1D (15). This distress not only affects parental well-being but also influences child outcomes, contributing to problematic behaviors (16) and further disruption of glycemic control (17). Parents often report fear of hypoglycemia and other diabetes-related complications, which can negatively affect the parent–child relationship (18). Moreover, less supportive parenting behaviors, such as criticism or overcontrol (19), are associated with increased psychological distress in children, leading to maladaptive behaviors (20) and suboptimal glycemic outcomes (21).

Lower parental self-efficacy has also been linked to increased levels of stress and depression, which are known to impact glycemic control negatively. These findings align with international recommendations that emphasize the importance of integrating routine psychological assessment into pediatric diabetes care (22). In addition, studies have shown that many children fail to achieve recommended HbA1c targets in clinical settings (23). Qualitative evidence further highlights parental challenges, including limited knowledge at diagnosis (24) and perceived inadequacies in diabetes care within school environments (25).

In Saudi Arabia, factors such as extended family members’ involvement and family network may provide emotional and practical support in managing T1D in children (26). Additionally, religious beliefs and coping practices play a crucial role in helping families cope with challenges related to chronic disease management (27). However, food-based cultural practices and social gatherings can make managing T1D in children even more challenging and lead to parents’ stress (28). Such factors highlight the significance of considering the role of stress and anxiety in parents within the Saudi culture compared to the Western population (29).

Despite growing recognition of the psychosocial dimensions of T1D management, there remains a lack of quantitative studies examining the relationship between glycemic control and parental distress and anxiety, particularly within the Saudi cultural context. The primary objective of this study was to examine the association between glycemic control and parental distress and anxiety among Saudi parents of children and adolescents with T1D.

## Materials and methods

### Study design and setting

The study was an analytical cross-sectional study conducted at the Pediatric Diabetes Clinic of King Fahd Specialist Hospital in Tabuk, Saudi Arabia, between January and June 2024. The data were gathered during regular follow-up visits, during which parents were interviewed, and the child’s latest laboratory-confirmed HbA1c level was extracted from the medical record. The Institutional Review Board of the University of Tabuk (UT-330-167-2023) provided ethical approval for the study, and all participants signed a written informed consent form.

### Study population and sampling strategy

The inclusion criteria included Saudi parents of children and adolescents with a confirmed diagnosis of T1D for at least six months, aged between 1 and 18 years. One parent per child was included. The exclusion criteria included non-Saudi parents, children diagnosed with type 2 diabetes, parents who refused to participate, and cases with incomplete questionnaire data or missing HbA1c values.

The convenience sampling method was used because the recruitment criteria relied on clinic attendance patterns. This method might introduce selection bias by favoring families who regularly attend follow-up visits, potentially leading to differences compared to those who do not. To minimize this limitation, all eligible parents attending the clinic on consecutive days were invited to participate.

A total of 87 parents participated in the study. The sample size was determined based on feasibility and comparison with similar cross-sectional studies on parental distress in pediatric T1D, which commonly include 70–120 participants. A *post hoc* power analysis indicated that the sample provided >80% power to detect medium effect sizes at α = 0.05.

### Measures

#### Glycemic control

HbA1c was analyzed both as a continuous measure and as a categorical variable. Children were classified as having good glycemic control (≤7.5%), intermediate glycemic control (7.6–9.0%), or poor glycemic control (>9.0%). These cutoffs are based on American Diabetes Association and ISPAD guidelines for pediatric glycemic targets. HbA1c values were used to assess glycemic control in medical records. Other glucose-measuring tools, such as CGM and CGM-derived metrics like TIR, were not used and therefore not included in the analysis.

#### Parental diabetes-related distress

Parental distress was assessed using a well-known standardized assessment method, “PDDS” (30), after translation into Arabic and cultural adaptation. It measures a total distress and four subdomains: personal distress, teen management distress, parent–teen relationship distress, and healthcare team distress. Previous studies have demonstrated strong reliability (α > 0.85). The PDDS comprises 20 items rated on a 5-point Likert scale ranging from 1 (not at all) to 5 (a great deal), with higher scores indicating greater diabetes-related distress. The scale has four domains: parental distress, teen management distress, parent-teen relationship distress, and healthcare team distress.

#### Parental anxiety

Another Arabic translation and cultural adaptation were conducted for the Spielberger State-Trait Anxiety Inventory State Anxiety subscale, a standardized assessment tool for parental anxiety (31). The STAI-State subscale was used to evaluate parental anxiety. It consists of 20 items on a 4-point Likert scale, with scores from 1 (not at all) to 4 (very much so). Total score range from 20 to 80, with high scores pointing to a greater anxiety level. One trained researcher conducted interviews, and a standardized protocol was used across all interviews to minimize interviewer bias. Information bias was minimized by using validated instruments and laboratory-confirmed HbA1c measurements.

### Statistical analysis

All analyses were conducted using SPSS version 27. The Shapiro–Wilk test was used to assess the normality of continuous variables. Normally distributed variables were summarized as mean ± standard deviation (SD), while skewed variables were summarized as median and interquartile range (IQR). Categorical variables were summarized as frequencies and percentages. For the duration of the disease, four categories were designed (<1 year, 1–3 years, 3–5 years, and >5 years). For facilitating statistical analysis and comparisons, these categories were redesigned into < 3years and ≥3 years for the sake of an adequate sample size within the group.

Comparisons of PDDS and STAI scores across HbA1c categories were performed using one-way analysis of variance (ANOVA). Associations between categorical variables and HbA1c categories were assessed using the chi-square (χ²) test. Cronbach’s α (acceptable ≥ 0.70) was used to assess the reliability of the PDDS and STAI. Pearson correlation coefficients were calculated to evaluate associations between HbA1c and psychological measures. Missing data were <5% and handled using pairwise deletion. A two-tailed p-value <0.05 was considered statistically significant.

## Results

**Figure 1:**
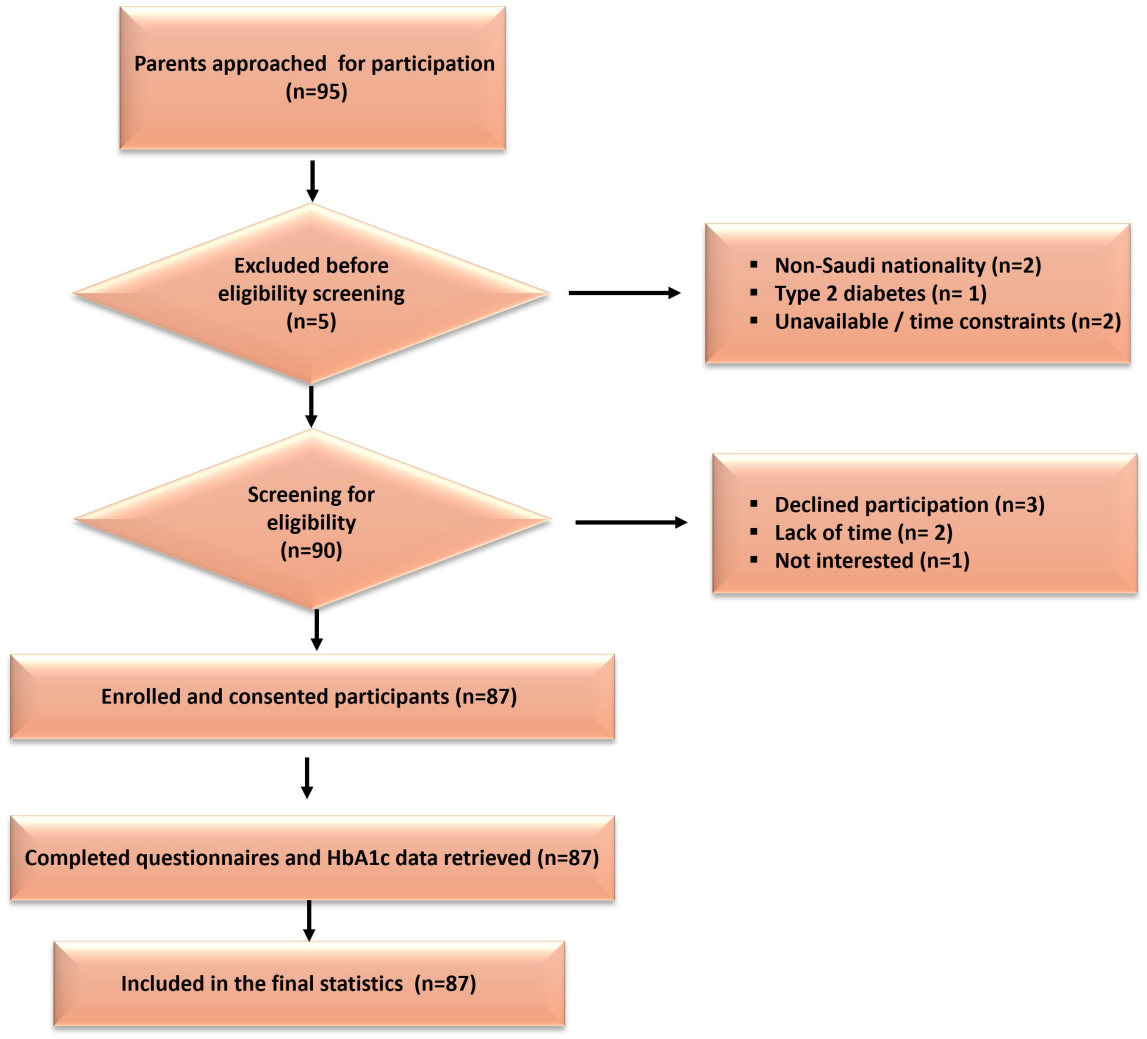
Flow diagram of participant recruitment and inclusion. A total of 95 parents were approached, of whom 90 met the eligibility criteria. Eighty-seven parents consented to participate and were included in the final analysis.

Of the 95 parents approached, 90 met the eligibility criteria, and 87 consented to participate (response rate: 96.7%). Three parents declined due to time constraints. All 87 participants completed the questionnaires and had valid HbA1c data; therefore, the final analysis included all participants with no missing data. A flow diagram summarizing participant recruitment and inclusion is presented in **Figure 1**.

Participant Characteristics: The mean age of children was 11.6 ± 4.2 years, and 51% were female. The median duration of diabetes was 3.8 years (IQR: 2.0–6.0). Parents had a mean age of 39.2 ± 7.8 years; 68% were mothers and 32% fathers.

### Glycemic control distribution and participant characteristics

The mean HbA1c level was 8.79 ± 1.73%. Based on established clinical cut-offs, 20.7% of children had good glycemic control (≤7.5%), 44.8% had intermediate glycemic control (7.6–9.0%), and 34.5% had poor control (>9.0%). Significant differences across HbA1c categories were observed for child sex (p = 0.04), age group (p = 0.01), diabetes duration (p = 0.02), and parental education (p = 0.04); however, the insulin regimen association with glycemic control was not significant (Fisher-Freeman-Halton exact test, p = 0.07) (**Table 1**).

**Table 1:** Distribution of glycated hemoglobin (HbA1c) categories according to child and parent characteristics (n = 87).

Variable	Good glycemic control (≤7.5%) n (%)	Intermediate glycemic control (7.6–9.0%) n (%)	Poor glycemic control (>9.0%) n (%)	p-value
Total sample	18 (20.7)	39 (44.8)	30 (34.5)	–
Sex				0.04
Male	6 (20.7)	13 (44.8)	10 (34.5)	
Female	12 (22.6)	26 (49.1)	15 (28.3)	
Age group (years)				0.01
<10	11 (36.7)	17 (56.7)	2 (6.6)	
10–15	6 (17.1)	16 (45.7)	13 (37.2)	
>15	1 (8.3)	4 (33.3)	7 (58.4)	
Insulin regimen				0.07 *
MDI	12 (16.4)	34 (46.6)	27 (37.0)	
BID	3 (27.3)	6 (54.5)	2 (18.2)	
Pump	3 (100.0)	0 (0.0)	0 (0.0)	
Disease duration				0.02
<3 years	8 (29.6)	13 (48.1)	6 (22.3)	
≥3 years	10 (17.9)	26 (46.4)	20 (35.7)	
Parental education				0.04
High school or less	6 (15.8)	15 (39.5)	17 (44.7)	
Bachelor’s or higher	12 (26.1)	24 (52.2)	10 (21.7)	

data presented as a number (percentage). HbA1c categories were defined according to standard clinical thresholds. Percentages are calculated within each row. MDI, multiple daily injections; BID, twice daily insulin injections. *P-values were obtained using the chi-square test, except for insulin regimen, for which the Fisher-Freeman-Halton exact test was used because of the small number of insulin pump users.

### PDDS and STAI scores across glycemic categories

Mean PDDS and STAI scores by HbA1c category are presented in **Table 2**. ANOVA showed no statistically significant differences in PDDS total scores across groups (p = 0.288), whereas STAI scores differed significantly by glycemic category (p = 0.004). Further sub-analyses examined the association between insulin regimen and parental anxiety, which showed no significant differences in parental anxiety. The mean ± SD STAI scores were 43.1 ± 7.0 for pump, 46.4 ± 7.5 for MDI, and 46.8 ± 7.3 for BID (p = 0.27).

**Table 2:** Parental diabetes distress (PDDS) and anxiety (STAI) scores according to glycated hemoglobin (HbA1c) categories (n = 87).

HbA1c category	n	PDDS score, mean ± SD	STAI score, mean ± SD
Good glycemic control (≤7.5%)	18	53.3 ± 20.7	30.7 ± 12.3
Intermediate glycemic control (7.6–9.0%)	39	61.2 ± 16.1	44.0 ± 14.2
Poor glycemic control (>9.0%)	30	60.7 ± 19.7	35.1 ± 16.6
p-value	—	0.288	0.004

Data presented as mean ± standard deviation (SD). P-values were obtained using one-way analysis of variance (ANOVA). PDDS, Parent Diabetes Distress Scale; STAI, State–Trait Anxiety Inventory.

### Parental diabetes distress subscale scores according to glycemic control

The PDDS subscale analysis showed a significant difference across the HbA1c categories. After Bonferroni correction for multiple comparisons, significant associations with glycemic control were teen management distress (adjusted p=0.04) and parent-teen relationship distress (adjusted p=0.004). In comparison, personal distress and healthcare team distress were not statistically significant (**Table 3**).

**Table 3:** Parent diabetes distress scale (PDDS) subscale scores according to glycated hemoglobin (HbA1c) categories (n = 87).

PDDS subscale	Good glycemic control, mean ± SD	Intermediate glycemic control, mean ± SD	Poor glycemic control, mean ± SD	p-value	Bonferroni-adjusted p-value
Personal distress	2.8 ± 0.6	3.1 ± 0.7	3.5 ± 0.8	0.02	0.08
Teen management distress	2.9 ± 0.5	3.3 ± 0.6	3.7 ± 0.7	0.01	0.04
Parent–teen relationship distress	3.0 ± 0.6	3.4 ± 0.7	3.8 ± 0.7	0.001	0.004
Healthcare team distress	2.7 ± 0.5	2.9 ± 0.6	3.2 ± 0.6	0.04	0.16

Data presented as mean ± standard deviation (SD). P-values were obtained using one-way analysis of variance (ANOVA). PDDS, Parent Diabetes Distress Scale. A Bonferroni-adjusted significance threshold of p < 0.0125 was applied for the four PDDS subscale comparisons.

### Sensitivity analysis

The omission of three extreme HbA1c values (>13%) did not significantly affect the results. Parent sex and PDDS or STAI scores did not show any significant interaction effects.

## Discussion

Our study showed that higher HbA1c levels associated with increased parental anxiety, while no statistically significant difference was observed for total diabetes-related distress across glycemic categories. Only 20.7% of children achieved the recommended baseline glycemic level (HbA1c ≤ 7.5%), highlighting overall suboptimal glycemic control in this cohort. This highlights the reported challenges in achieving optimal glycemic control among Saudi pediatric patients with T1D. Insufficient diabetes education programs, specifically carbohydrate control, limited family support, and the school setting, and other factors may contribute to suboptimal glycemic outcomes (32).

The relationship between HbA1c levels and parental psychological factors appears complex and multidimensional. Higher HbA1c levels were associated with increased parental anxiety. This observation is consistent with previous literature indicating that parents of children with chronic conditions, such as T1D, often experience sustained vigilance and fear of acute and long-term complications (18, 33). Given the study’s cross-sectional design, a direct relationship cannot be established. While parental anxiety may increase challenges in managing the diabetes of their child or adolescent, persistently raised levels of HbA1c and worries about complications of diabetes may increase stress and anxiety in parents (34). Within domains, the greatest association was found for the teen management distress and parent/teen relationship distress. After adjustment for multiple comparisons, however, these two domains remained significantly associated with glycemic control; thus, demonstrating that diabetes management and parent/adolescent relationships are most strongly associated with the adolescent’s glycemic control (34). From a developmental perspective, the increasing autonomy and independence in diabetes self-management during adolescence create tension between adolescents seeking greater responsibility for diabetes and parents who remain concerned about diabetes treatment and glycemic control. The presence of such diabetes-related distress and parent-teen conflict may adversely influence glycemic outcome (35).

The study showed that the PDD subscale was more sensitive to glycemic control-related challenges than the total PDDS score. Therefore, a domain-specific assessment may be valuable in identifying families who could benefit from targeted psychological intervention. These findings align with evidence showing that domain-specific aspects of parental distress are closely linked to diabetes management behaviors and glycemic outcomes in children with T1D (16). In addition to parent-adolescent conflict factors, the psychological burden associated with advanced diabetes technologies should not be overlooked (36). As continuous glucose monitoring and insulin delivery systems have improved glycemic outcomes, data overload and alarm fatigue have been described in the literature as a psychological burden for people with diabetes, caused by the large amounts of real-time data and frequent alerts from glucose-monitoring and insulin-injection devices (37).

Notably, older age and longer disease duration were associated with poorer glycemic control, aligning with broader clinical observations and existing international evidence (11, 12). The older a child is, the greater the associated autonomy, which may create challenges in maintaining optimal diabetes management (38). Another strain during this developmental period is the significant parent–teen relationship distress. This culminates in a cyclical chain of events that further disrupts self-management and glycemic control among adolescents, and is exacerbated by parental concern and conflict (25, 39). Moreover, longer diabetes duration and impaired glycemic control may reflect treatment fatigue, in which ongoing disease management becomes burdensome and may affect adherence (28, 32). Taking into consideration psychological changes during adolescence, such as increased insulin resistance and parental-adolescent conflict, both of which may be associated with poor glycemic control, in addition to old age and long disease duration.

A positive finding of the present study was that higher parental education may act as a protective factor, supporting better glycemic outcomes. Higher education may empower parents with better understanding and management skills (39). This may enhance parents’ ability to navigate healthcare systems and support effective disease management. These findings are of utmost importance, as diabetes-related education should be accessible to every family member, regardless of their disease status or educational level. Beyond parental education, Saudi extended and/or family networks may influence the practical and emotional support needed to alleviate the burden of the parent with diabetes and their other family members. However, families with children with diabetes can experience increased parental stress from the family’s expectations regarding diabetes care and parenting in general (40).

On contrast, parental anxiety did not significantly differ between the insulin regimens in this study. Although the average anxiety score for parents of children on a pump was numerically lower than for children on MDI or BID regimens, the number of children on a pump in this study was too small to draw any meaningful conclusions between the different insulin regimens. The small number of people using insulin pumps in this cohort may reflect insufficient use and access to diabetes technologies. Recent data from Saudi studies showed that insulin pumps and continuous glucose monitoring systems (CGMS) use may affect psychological well-being and diabetes-related burden in the T1D Saudi population (41). However, as this only applies to three of the participants using insulin pumps, the study could not provide adequate information regarding the impact of such devices on parental anxiety.

Despite these insights, our study has several limitations. The cross-sectional design limits causal inference: therefore, only associations, not causation, can be inferred, and the relationship between glycemic control and parental distress may be bidirectional (16, 17). The generalizability of our findings may be limited by the single-center design and relatively small sample size. Multicenter studies with larger and more diverse populations are needed to confirm the observed associations and enhance external validity. Additionally, reliance on self-reported measures may introduce reporting bias.

Further limitations include the measurement of anxiety performed using the state anxiety scale as a part of the State Trait Anxiety Inventory (STAI). While such a test allows measuring anxiety at present, it does not measure long-standing anxiety. Future studies involving both state and trait anxiety may demonstrate a comprehensive parent’s psychological burden. The socioeconomic factors were not comprehensively evaluated, as only parents’ education was evaluated. In addition, parental comorbid psychiatric disorders were not assessed. Such factors may affect both parental psychological burden and glycemic outcomes and need to be considered in future studies. Data from CGM and CGM-derived metrics were unavailable, limiting a full assessment of glycemic variability beyond HbA1c.

Our findings have important clinical implications. Psychological screening should be integrated into routine care for children with type 1 diabetes, particularly for families at risk of emotional distress. Interventions with a purely psychosocial focus should shift from general emotional support to include strategies to build skills in diabetes management, communication, and parent-adolescent collaboration, thereby addressing the sources of distress that most directly affect the patient’s metabolic control. Early identification of parental distress may allow timely referral to psychological support services and targeted educational programs. In addition, culturally tailored, family-based interventions are needed to improve communication, support diabetes management, and enhance both emotional well-being and glycemic outcomes. While regional data on the psychosocial burden of pediatric T1D are limited, our findings provide evidence for integrating family-centered care into routine diabetes services. In addition, assessing parents’ psychological well-being and offering them support can help parents with high anxiety and parents of children with poor glycemic control. A combination of both PDDS and STAI delivered complementary data regarding diabetes related distress and anxiety may represent a useful practical screening approach in a pediatric diabetes clinic. However, while the ideal screening frequency remains unclear, a periodic assessment during diabetes clinic follow-up may accelerate early identification of families experiencing significant psychological burden and promote timely intervention.

To conclude, poor glycemic control in children and adolescents with T1D is linked to parental distress and anxiety. This study provides notable regional evidence gaps, highlighting the importance of parental psychological well-being for glycemic control among Saudi children and adolescents with T1D. The finding emphasizes the need to integrate periodic psychological assessment and family-centered, targeted psychological support into the diabetes care clinic. Future longitudinal and interventional studies are warranted to determine whether targeting parental distress may improve glycemic outcomes and overall family functioning.

## Data Availability

The original contributions presented in the study are included in the article/supplementary material. Further inquiries can be directed to the corresponding author.
